# The prohibitins (PHB) gene family in tomato: Bioinformatic identification and expression analysis under abiotic and phytohormone stresses

**DOI:** 10.1080/21645698.2021.1872333

**Published:** 2021-03-08

**Authors:** Feiyan Huang, Xianwen Ye, Zhijiang Wang, Yan Ding, Xianjie Cai, Lei Yu, Muhammad Waseem, Farhat Abbas, Umair Ashraf, Xiaolong Chen, Yanguo Ke

**Affiliations:** a College of Agriculture and Life Sciences, Yunnan Urban Agricultural Engineering & Technological Research Center, Kunming University Kunming, China; b Kunming Tobacco Corporation of Yunnan Province, Kunming, China; c Material Procurement Center, Shanghai Tobacco Group Co., Ltd, Shanghai, China; d State Key Laboratory for Conservation and Utilization of Subtropical Agro-Bioresources, College of Horticulture, South China Agricultural University, Guangzhou, China; e The Research Center for Ornamental Plants, College of Forestry and Landscape Architecture, South China Agricultural University, Guangzhou, China; f Department of Botany, Division of Science and Technology, University of Education, Lahore, Pakistan; g Tobacco Leaf Purchase Center, China Tobacco Henan Industrial Co., Ltd, Zhengzhou, China; h College of Economics and Management, Kunming University, Kunming, China

**Keywords:** Tomato, prohibitins, stress, phylogeny, synteny, expression

## Abstract

The prohibitins (PHB) are SPFH domain-containing proteins found in the prokaryotes to eukaryotes. The plant PHBs are associated with a wide range of biological processes, including senescence, development, and responses to biotic and abiotic stresses. The PHB proteins are identified and characterized in the number of plant species, such as *Arabidopsis*, rice, maize, and soybean. However, no systematic identification of PHB proteins was performed in *Solanum lycopersicum*. In this study, we identified 16 PHB proteins in the tomato genome. The analysis of conserved motifs and gene structure validated the phylogenetic classification of tomato PHB proteins. It was observed that various members of tomato PHB proteins undergo purifying selection based on the Ka/Ks ratio and are targeted by four families of miRNAs. Moreover, SlPHB proteins displayed a very unique expression pattern in different plant parts including fruits at various development stages. It was found that SlPHBs processed various development-related and phytohormone responsive *cis*-regulatory elements in their promoter regions. Furthermore, the exogenous phytohormones treatments (Abscisic acid, indole-3-acetic acid, gibberellic acid, methyl jasmonate) salt and drought stresses induce the expression of SlPHB. Moreover, the subcellular localization assay revealed that SlPHB5 and SlPHB10 were located in the mitochondria. This study systematically summarized the general characterization of SlPHBs in the tomato genome and provides a foundation for the functional characterization of PHB genes in tomato and other plant species.

## 1. Introduction

The prohibitins (PHB) genes concede highly conserved stomatin/prohibitin/flotillin/HflK/C (SPFH) domain in their protein sequence also recognized as band_7 domain proteins.^1^ PHBs proteins are ubiquitous proteins and are associated with a variety of biological processes including cell cycle, apoptosis, and respiration.^2–5^ PHBs have been identified from eukaryotes, fungi, plants, and animals.^6,7^ In humans, the PHB proteins act as transcriptional regulators interacting with *PSF3*, retinoblastoma proteins (*Rb*), and *E2F*.^8,9^ PHB genes were observed to be linked with the breast cancer phenotype, where they localize in the nucleus of breast cancer cell lines as a transcriptional regulator via interaction with P53, RB and E2F to regulate the expression of downstream genes. PHBs were also identified in lipid raft, a key constituent of cell membrane.^10–13^ Similarly, PHBs found in plasma membrane were considered to act as a target for small molecules in the inflammatory reponses as well as to regulate the membrane receptor and iron channels.^14,15^ In short, PHB genes play crucial roles in different biological processes and are associated with various disease phenotypes. However, less is known about the role of PHB proteins in the plant kingdom.

PHB proteins are classified into type I and type II and both are complimentary for stability and functioning of PHB protein.^17^ In mammals, *PHB1* and *PHB2* have been well characterized and shown to form a 1–2 KDa protein complex on the inner mitochondrial membrane. In addition, the absence of any of these two proteins failed to produce this protein complex in *Caenorhabditis elegens*, resulted in decreased PHB proteins. PHB complex have been physically and functionally linked with the matrix-ATPase related to diverse cellular activities (m-AA) to regulate the degradation of respiratory chain proteins in mitochondria.^18^ PHB and PHB2/REA were found to be involved in maintaining cellular survival via Ras–Raf–MEK–Erk pathway.^19^ These findings suggest that both types of PHB are required for stable complex formation and proper functioning.^5,20,21^Recently, various studies reported the role of PHB in plants. These proteins play a pivotal role not only in plant development and senescence but also in responses to abiotic and biotic stresses.^22,23^ *PHB3* and *PHB4* are the most broadly studied PHB genes from *Arabidopsis thaliana*, where they primarily expressed both in root and shoot proliferative tissues. *Arabidopsis* mutant, *atphb3* exhibited severely retarded growth phenotypes, decreased stem, root proliferation, and declined cell division in root and stem apices.^24^ Overexpression of *Arabidopsis* PHB (*AtPHB3/AtPHB4*) exhibited irregular leaf shape and extensive branching phenotype.^24^ Notably, *atphb3*/4 double knockout mutants were not viable, suggesting that PHBs play important role in plant development.^24^ Similar results were obtained in petunia and tobacco, where PHB-silenced genes showed decreased cell production and prolonged senescence.^25,26^ In tobacco, suppression of *NbPHB2* delays growth and promotes leaf senescence and apoptosis.^26^ Moreover, the cells in silenced flowers were larger as compared to control flowers, suggesting a significant decrease in the number of cell division that occurs during corolla development. PHB proteins directly or indirectly interact with mitochondrial DNA (mtDNA) to regulate the reactive oxygen species (ROS) formation and oxidative phosphorylation (OXPHOS), which potentially lead to senescence phenotype both in *C. elegans* and plants.^20,25–27^ Furthermore, PHB protein might also involve in maintaining crista morphology to employ proteins into the inner membrane.^21,28^ The abovementioned finding indicates that PHB play key functioning in cell proliferation. Several studies have shown that PHB proteins play key roles not only in plant development and senescence but also in response to salinity, defense and plant hormones. For instance, *Arabidopsis eer3-1*(*atphb3*) mutant showed an etiolated seedling phenotype upon constitutive exposure to ethylene with suppressed the expression of various ethylene inducible genes (Arabidopsis ethylene-responsive element binding protein [AtEBP], plant defensin [PDF 1.2]), indicating the dual role of *AtPHB3* in *Arabidopsis*.^29^ Additionally, AtPHB3 acted downstream of ethylene insensitive 2 (*EIN2*) and *EIN3*. A loss of function mutant *atphb3-3* failed to affect diverse biological processes such as nitric oxide (NO) signaling, ABA (abscisic acid) induced stomatal closure, IAA (auxin) induced root formation.^30^ This mutant resulted from the substitution of Gly at position 165 with Asp of *AtPHB3* protein. However, another *Arabidopsis* PHB (*At5g64870*) induced under cold, salinity, and drought but suppressed in response to hormones such as gibberellin (GA), methyl jasmonate (MeJA), and ABA.^31^ PHB proteins have been identified in various plant species including 17 in *Arabidopsis*, 19 in rice,^31^ 24 in *Glycine max*,^32^ and *Zea mays* with 16.^17^ The knowledge about PHB genes in tomato is insufficient. In this study, a total of 16 PHB genes were identified in the tomato genome. Phylogenetic analysis, gene structure, *in silico* subcellular location prediction, *cis*-regulatory elements, MEME motif scan, and protein chromosome location were also conducted. In addition, tissues/organ-specific expression profiling under normal conditions was evaluated. Moreover, differential expression patterns under salt, drought, and hormone-induced expression were analyzed. This study enables us to provide a foundation for the functional characterization of PHB genes in tomato.

## 2. Material and Method

### 2.1. The Tomato PHB Gene Discovery

To predict PHB genes in the tomato genome, the *Arabidopsis*, rice, *Zea mays*, and *Glycine max* PHB peptide sequences were retrieved from the TAIR genome database (https://www.arabidopsis.org/),^33^ rice genome annotation project (http://rice.plantbiology.msu.edu), phytozome database (https://phytozome.jgi.doe.gov/), respectively. These sequences were used as a query in the SOL genome network (https://solgenomics.net).^34^ The candidates’ sequences were analyzed for SPFH Domain (PF01145) in the SMART (http://smart.embl-heidelberg.de)^35^ and NCBI conserved domain database (CDD, https://ncbi.nlm.nih.gov/Structure/bwrpsb/bwrpsb.cgi).^36^ Moreover, PHB protein features including the isoelectric point (pI), the grand average of hydropathy (GRAVY), molecular weight (kDa) of each protein were calculated in sequence manipulation suite (SMS, bioinformatics.org/sms2).^37^ The deduced PHB proteins were named in their order on the tomato chromosomes.

### 2.2. Phylogenetic Analysis and Ka/Ks Analysis of Duplications

Clustal Omega (ClustalO, https://www.ebi.ac.uk/Tools/msa/clustalo/)^38^ program was used to generate SlPHB peptide sequences alignment. For the phylogenetic relationship, SlPHBs peptide sequences from rice, *Arabidopsis, Zea mays*, and soybean were retrieved from phytozome (https://phytozome.jgi.doe.gov). An unrooted neighbor-joining^39^ tree was constructed using MEGAX software^40^ with the parameters set as follows: Poisson correlation of model; pairwise deletion of gaps/missing data; random seed of phylogeny test and bootstrap was set at 1000 replicates. The non-synonymous (Ka), synonymous (Ks) nucleotide substitution rates and the Ka/Ks ratio were predicted using k-estimator (http://en.bio-soft.net/format/KEstimator.html).^41^ The divergence time (T) was calculated as follows: T = Ks/2y (y = 6.56 x 10^−9^).^42^

### 2.3. Chromosome Location, Subcellular Location Prediction, and miRNA Target Prediction

The chromosome position of each SlPHB gene was obtained from the SOL genome and visualized in the MAPGene2Chromsome program (http://mg2c.iask.in/mg2c_v2.0/). *In silico* subcellular location, prediction analysis was performed in the WoLFPSORT program (https://wolfpsort.hgc.jp).^43^ To predict miRNAs targeted putative PHBs, the cDNA sequences of each *SlPHBs* were submitted to psRNATarget^44^ against all tomato miRNAs reported in miRbase.^45^

### 2.4. *Gene Structure Analysis, Conserved Motif Scan, and* Cis*-Regulatory Motif Prediction*

The retrieved tomato *SlPHBs* coding sequences (CDS) and genomic sequences were submitted to the Gene Structure Display Server (GSDS, http://gsds.cbi.pku.edu.cn)^46^ for intron and exon distribution in each gene. MEME suite (http://meme-suite.org)^47^ was used to predict conserved motifs in SlPHB protein sequences with a parameter set as follows: (i) a maximum number of motifs – 10, (ii) number of repetitions – any, (iii) optimum motif width set to ≥10 and ≤50. A 1000bp 5`UTR nucleotide sequences from the start codon (ATG) of each SlPHB gene were retrieved from the SOL genome and scanned in the PlantCRAE database (http://bioinformatics.psb.ugent.be/webtools/plantcare/html/)^48^ for cis-regulatory elements prediction.

### 2.5. Plant Material, Abiotic Stress, and Phytohormone Treatment

Tomato cv. Micro-Tom seedlings were grown in the College of Agriculture and life sciences, Kunming University, under controlled greenhouse conditions (25°C/20°C, day/night, 14 h/10 h light/dark photoperiod with relative humidity 80%). For tissue/organ-specific expression analysis of various plant parts such as root, leaves, stem, and flowers were collected from a six-week-old plant. For expression in fruit tissues, 1/2/3/cm, mature green fruit, breaker fruit, and ten days breaker fruits were harvested.^49^ For salinity, drought, and phytohormone-induced stresses, six-week-old plants were treated with 200 mM NaCl, 0.01 mM ABA, GA3, IAA, MeJA, and PEG as described previously.^50^ Roots and shoots (including stem and leaves) were harvested at 0 h, 3 h, 6 h, 12 h, and 24 h interval after treatment. All the samples were collected in triplicate and store immediately at −80°C.

### 2.6. Total RNA Extraction, cDNA Preparation, and qRT-PCR Analysis

Total RNA was extracted from selected samples using TRIZOL reagent according to the manufacturer’s instruction. RNA was quantified using nanodrop lite (Thermo USA) and RNA integrity was assessed by running 2% agar agarose gel electrophoresis. The cDNA was synthesized with a PrimerScript Real-Time (RT) reagent kit (Takara, Japan) according to the manufacturer’s protocol as described previously.^51–53^ RT-qPCR was conducted in ABI 7500 Fast Real-Time system (AB, USA) using the iTaq™ Universal SYBR® Green Supermix (BIO-RAD, USA) according to the manufacturer’s protocol. The RT-qPCR was conducted in triplicate. Tomato *SlUBQ* (*Solyc01g056940*) gene was used as an internal control. The relative expression of tomato *SlPHBs* was calculated using the 2^−ΔΔCt^ method^54^ and heat maps were generated with heat mapper program (http://www1.heatmapper.ca/expression/).

### 2.7. Subcellular Localization of SlPHB5 and SlPHB10

The full-length sequences of SlPHB5 and SlPHB10 excluding stop codon were fused into the vector p35S-GFP as explained previously.^51,55^ The Arabidopsis protoplast isolation and transformation were carried out as described by Sheen.^56^ After 18–20 h of transformation, the protoplast was visualized by confocal laser scanning microscope and the images were processed using photoshop.

## 3. Results

### 3.1. Identification of SlPHB Genes

**Figure 1.**
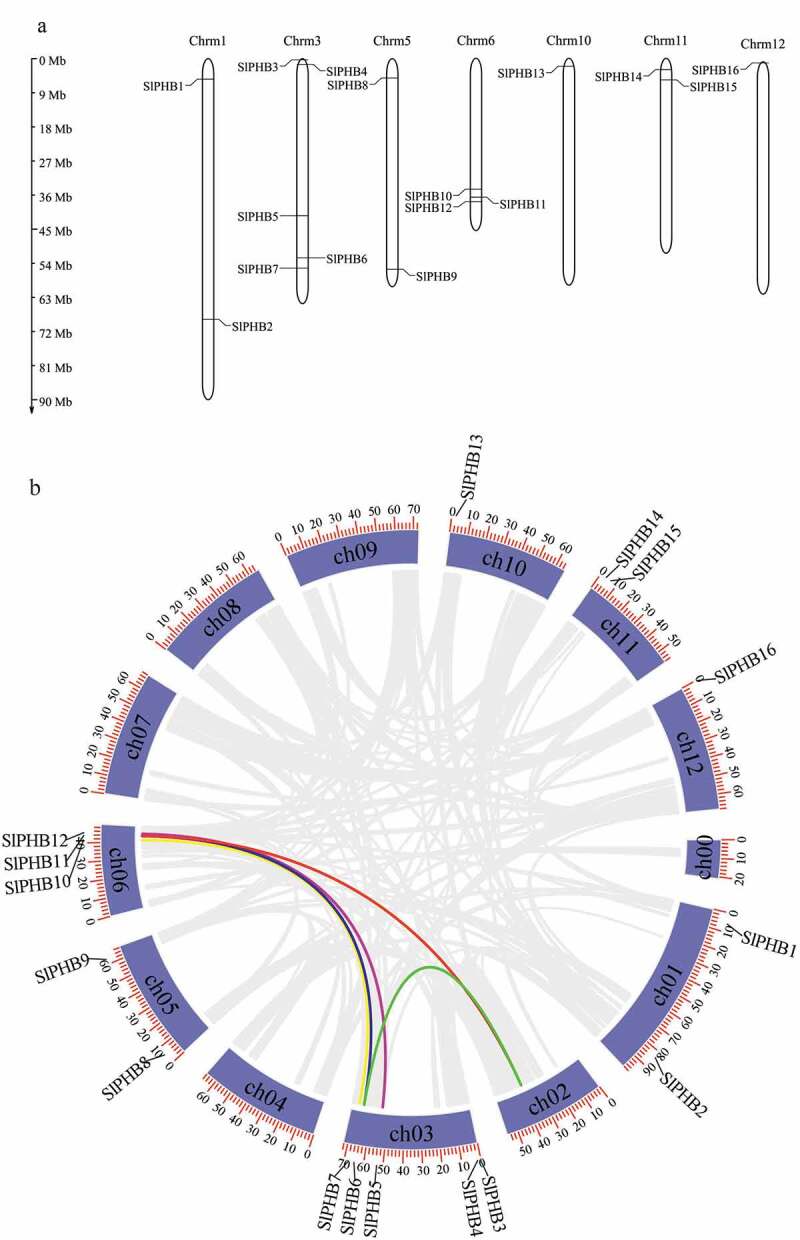
Chromosomal location and synteny of PHB genes in the tomato genome. (a) The chromosome location of tomato SLPHB genes. The scale of chromosomes is in megabases (MB). (b) Circos plot presenting gene segmental duplication events of PHB genes. Segmental duplication pairs are indicated with different color lines.

The Arabidopsis, rice, *Zea mays*, and *glycine max* PHB protein sequences were used as a query in the SOL genome to identify all putative PHB protein sequences in the tomato genome. A total of 16 non-redundant genes were identified. The Pfam, SMART, and NCBI CDD searches were used to verify the SPFH domain in all SlPHBs protein sequences. The tomato PHB genes were named as *SlPHB1* to *SlPHB16* in order of their position in chromosomes. The peptide length to the molecular weight of *SlPHBs* ranged from 261 aa (*SlPHB8*) to 518 aa (*SlPHB7*), and 30.08 kDa (*SlPHB1*) to 57.75 kDa (*SlPHB7*). The GRAVY values of all the *SlPHB* proteins were negatively exhibiting indicating that these proteins are hydrophilic except *SlPHB15* (Solyc11g013260) which show a positive GRAVY score. The deduced *SlPHB* genes were distributed in seven chromosomes (Fig. 1(a)). A pair of genes *SlPHB1* and *SlPHB2, SlPHB8* and *SlPHB9, SlPHB14*, and *SlPHB15* were located on chromosomes 1, 5, and 11 each, respectively. *SlPHB3, SlPHB4, SlPHB5, SlPHB6*, and *SlPHB7* were located on chromosome 3. Three genes (*SlPHB10, SlPHB11, SlPHB12*) were located on chromosome 6 while a single gene was located on chromosome 10 (*SlPHB13*) and chromosome 12 (*SlPHB16*) each. *In silico* subcellular location, prediction indicated that SlPHBs were localized in the cytoplasm, mitochondria, and chloroplast (Table 1). Tomato PHB genes displayed segmental duplication and five segmental gene duplication (eight genes) were found in tomato as shown in Fig. 1(b).

**Table 1.** The characteristic features of tomato SlPHB proteins in tomato genome

Gene locus ID	Gene Name	aa	MW	pI	GRAVY	Chromosome			Sub-cellular Location
						Position	Start	End	
Solyc01g010770	SlPHB1	272	30.08	4.55	−0.118	1	5825560	5828696	Cysk
Solyc01g089910	SlPHB2	490	54.12	7.22	−0.308	1	75383651	75385711	Cyto
Solyc03g005420	SlPHB3	489	54.67	8.99	−0.419	3	296785	298965	Cyto
Solyc03g007190	SlPHB4	290	32.32	4.89	−0.234	3	1764428	1769612	Cyto
Solyc03g080050	SlPHB5	424	46.49	9.48	−0.302	3	45473113	45480181	Mito
Solyc03g113220	SlPHB6	285	31.36	5.73	−0.087	3	57486387	57489392	Cyto
Solyc03g117250	SlPHB7	518	57.75	6.25	−0.389	3	60489302	60492373	Cyto
Solyc05g012340	SlPHB8	261	41.09	6.73	−0.377	5	5601824	5605948	Chlo
Solyc05g051510	SlPHB9	277	30.31	7.67	−0.016	5	61017078	61019992	Extr
Solyc06g065850	SlPHB10	484	53.94	5.26	−0.402	6	37670367	37673877	Mito
Solyc06g071050	SlPHB11	289	31.82	5.22	−0.119	6	40043522	40046056	Cyto
Solyc06g073030	SlPHB12	398	44.54	9.14	−0.429	6	41388909	41390901	Chlo
Solyc10g008140	SlPHB13	289	31.83	10.11	−0.154	10	2276303	2278610	Chlo
Solyc11g010190	SlPHB14	279	30.68	9.4	−0.057	11	3269915	3270754	Chlo
Solyc11g013260	SlPHB15	301	32.95	9.55	0.059	11	6170897	6173581	Chlo
Solyc12g005500	SlPHB16	283	31.16	10.08	−0.201	12	293644	295414	Chlo

aa; amino acid, MW; molecular weight, pI; isoelectric point, GRAVY; the grand average of hydropathy, Cysk; cytoskeleton, Cyto; cytoplasm, Chlo; Chloroplast, Mito; mitochondria, Extr; extracellular cytoplasm.

### 3.2. Phylogeny, Strong Purifying Selection, and Conserved Motif Analysis of SlPHB Proteins

**Figure 2.**
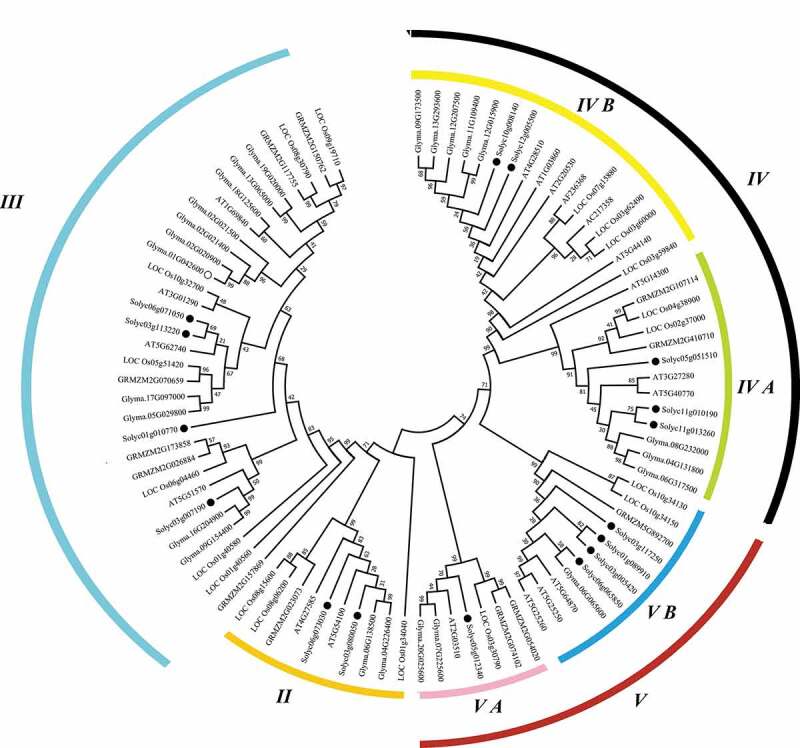
The phylogeny of the PHB proteins. An unrooted neighbor-joining phylogenetic tree of PHB proteins from *Arabidopsis*, rice, maize, soybean, and tomato was generated in the MEGA program with a bootstrap value set as 1000 replicates. The tree was clustered into various clades and subclades. The black dots represent tomato SlPHB proteins.

**Figure 3.**
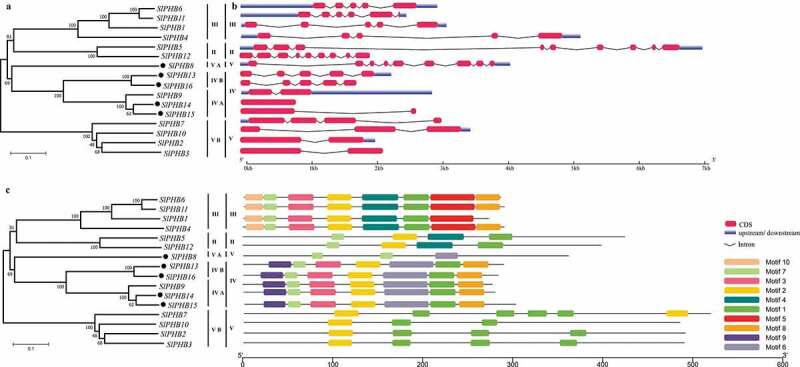
Phylogeny, gene exon/intron distribution, and conserved motif analysis of 16 tomato SlPHB genes. (a) An unrooted neighbor-joining phylogenetic tree of PHB proteins with bootstrap set at 1000 replicates and clustered into different clades and subclades. (b) Tomato SlPHB gene intron and exon distribution. The scale at the bottom is corresponding to gene size in kb. (c) The putative conserved motifs in 16 tomato PHB proteins identified using the MEME suite. A total of ten motifs (1 to 10) were identified and each color of the box is corresponding to a motif. The scale at the bottom represents the protein size in kb.

To unveil the phylogenetic relationship of tomato SlPHB proteins with PHBs from other plant species such as *Arabidopsis*, rice, maize, and soybean, an unrooted neighbor-joining phylogenetic tree was generated. It was observed that all PHB proteins were divided into four major clades (II, III, IV, and V). The subclade of each group contains 7–15 members from different species. SlPHBs were found in all clades such as five SlPHBs in group IV (2 in IV B and 3 in IV A). Similarly, three in subclade V B and single in V A subclade of major clade V. Moreover, clade III has four, and clade II contained two tomato SlPHBs. Similar trends of PHB distribution were observed for other species (Fig. 2). Furthermore, three sister pairs of SlPHB genes were detected in the phylogenetic tree such as *SlPHB14/SlPHB15* in clade IV A, *SlPHB2/SlPHB3* in subclade V B of major clade V, and *SlPHB11/SlPHB6* in clade III. It was observed that SlPHBs localized in chloroplast were clustered together as shown in Fig. 3(a).

A comparison of the gene structure of each tomato PHB revealed a diverse structure. The number of intron and exon ranged from one to nine exons and zero to eight introns. The exon/intron pattern was similar in different clades and subclades. For example, five exon and four introns were found in clade III, nine exons and eight introns in clade II, and clade V. Similarly, five exons in subclade IV B and two in IV A clade. Besides, the length and positions of exons were also highly similar in clades and subclade Fig. 3(b). We identified ten conserved motifs in SlPHBs using the MEME server. It was observed that the motifs pattern was also similar within clades (Fig. 3(c)). For instance, motif 1 and motif 2 found in clade V B; motif 1, motif 2, motif 3, motif 7, motif 8, and motif 9 in clade IV. SlPHBs in clade III contained all motifs except motif 9. To explore the fate of divergence of these genes in the tomato genome, the Ka/Ks values were estimated for three duplicate SlPHB gene pairs. The Ks was used in estimating the divergence time of each SlPHB gene pairs (Table 2). Our results showed that the Ka/Ks ratio of duplicated genes pairs was more than 0.04. This suggesting that the purifying selection pressure was a major factor that occurs during the evolution, function divergence was limited after duplication and was estimated to occur between 36.8 and 101.55 million years ago (Mya).

**Table 2.** The Ka/Ks of tomato SlPHB paralogs

Gene1	Gene2	Ka	Ks	Ka/Ks	Time (Mya*)	Purify Selection
Solyc01g089910	Solyc03g005420	0.206309991	1.247723962	0.165349065	95.1009117	Yes
Solyc03g113220	Solyc06g071050	0.042588715	0.483257784	0.088128358	36.83367252	Yes
Solyc11g010190	Solyc11g013260	0.067198744	1.332405685	0.050434147	101.5553114	Yes
*millions year ago						

### 3.3. Bioinformatics Analysis of SlPHB Promoter Sequences

**Figure 4.**
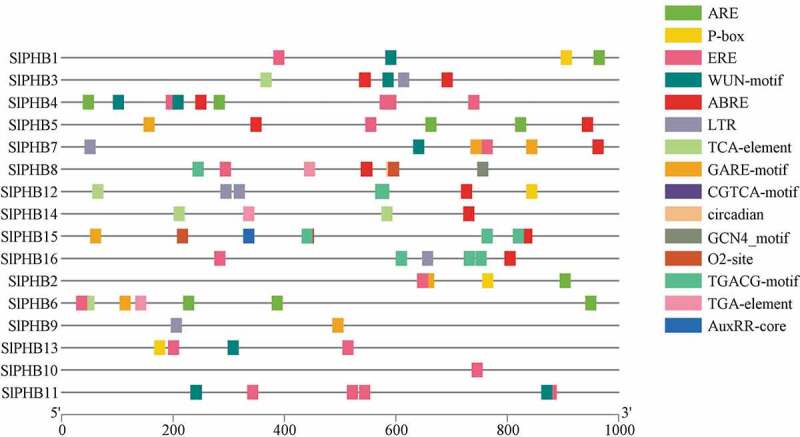
The putative *cis*-regulatory sequences were identified in 16 tomato *SlPHB* genes by submitting their corresponding promoter sequences to the PlantCARE database. Different *cis*-regulatory elements circadian control (circadian), meristem development (CAT-box), endosperm development (GCN4_motif), zein metabolism regulation (O2-site), MYB binding site involved in drought-inducibility (MBS), WRKY binding site involved in abiotic stress and defense response (W-box), anaerobic induction element (ARE), defense- and stress-responsive element (TC-rich repeats), low-temperature-responsive element (LTR), wound-responsive element (WUN-motif), element for maximal elicitor-mediated activation (AT-rich sequence) ethylene (ERE), gibberellin (GARE-motif), methyl jasmonate (MeJA, CGTCA-motif), abscisic acid (ABRE), and salicylic acid (TCA-element) and son on was detected.

The *cis*-acting elements of potential tomato SlPHB genes were predicted by searching a 1000 bp region from the transcriptional activation site (ATG) of each gene against the PlantCARE database. As shown in Fig. 4, several putative *cis*-regulatory sequences were identified in *SlPHB* genes. For an instance, four different kinds of development-related *cis*-regulatory elements such as circadian control (circadian), meristem development (CAT-box), endosperm development (GCN4_motif), and zein metabolism regulation (O2-site) were predicted in the promoter region of some of the SlPHBs, suggesting that these genes may play roles in organ/tissue-specific development and growth. Moreover, a various stress-responsive element such as the MYB binding site involved in drought-inducibility (MBS), WRKY binding site involved in abiotic stress and defense response (W-box), anaerobic induction element (ARE), defense- and stress-responsive element (TC-rich repeats), low-temperature-responsive element (LTR), wound-responsive element (WUN-motif), and element for maximal elicitor-mediated activation (AT-rich sequence) were also detected. The promoters of tomato SlPHB genes possessed *cis*-regulatory sequences related to ethylene (ERE), suggesting that these genes may involve in ethylene responses (Fig. 4). In addition, various hormone-related responsive elements related to gibberellin (GARE-motif), methyl jasmonate (MeJA, CGTCA-motif), abscisic acid (ABRE), and salicylic acid (TCA-element) were also detected, implying that these genes may respond to phytohormone as well (Fig. 4). The promoters of tomato *SlPHB* genes possessed *cis*-regulatory sequences related to ethylene (ERE), suggesting that these genes may involve in ethylene responses.

### 3.4. miRNAs Targeting the PHB Family Members of the Tomato

To find out miRNAs targeting the SlPHBs of tomato, the sequences were subjected to the miRNA database. The psRNATarget predicted that four SlPHBs gene family members were targeted by conserved miRNAs belongs to different miRNAs gene families each. *SlPHB7* was targeted by the sly-miRNA869 family and sly-miRNA4239 cause the cleavage of *SlPHB3*. A single member from sly-miR396 and sly-miR397 family member target to cleavage of *SlPHB15* and *SlPHB13* gene, respectively (Table S1).

### 3.5. Expression Analysis of SlPHB Genes in Different Plant Parts

**Figure 5.**
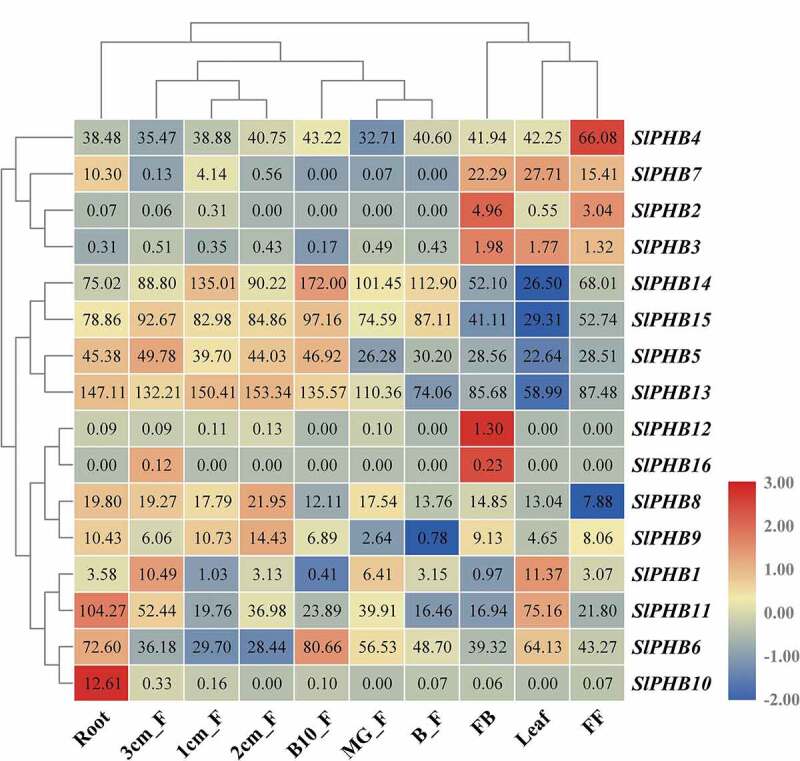
The endogenous expression profile of 16 tomato *SlPHB* genes in various plant parts including root, leaves, FB (flower bud), FF (fully opened flower), 1/2/3 cm fruit, mature green fruit (MG_F), breaker fruit (B_F), and 10 days breaker fruit (B10_F). A log2 transformed heatmap was generated using heatmapper program. Blue, white, and red color is corresponding to low, moderate, and high expressions. The genes were clustered by applying the Euclidean method.

To understand the role of putative *SlPHBs* in tomato plant growth and development, the expression profile analysis of *SlPHBs* in various plant parts was evaluated. The *SlPHBs* exhibited a diverse expression pattern among various plant parts. It was found that two *SlPHBs* were expressed in leaves, and root tissues. One *SlPHB* gene had high expression levels in fully opened flower and three expressed in flower at bud condition. It was observed that the number of genes was expressed in fruit at different development stages with more and less expression levels. For example, *SlPHB1* in 3 cm fruit, *SlPHB6* in ten days fruit breaker, *SlPHB8*, and *SlPHB9* in 2 cm fruit. However, *SlPHB5, SlPHB14*, and *SlPHB15* exhibited increasing expression during fruit development and ripening (2 cm fruit till ten days breaker fruit) (Fig. 5). The results showed that tomato *SlPHB* genes play an important role in the growth and development of specific plant parts or tissues.

### 3.6. Expression Profile of Tomato SlPHB in Response to Salinity and Drought Stress

**Figure 6.**
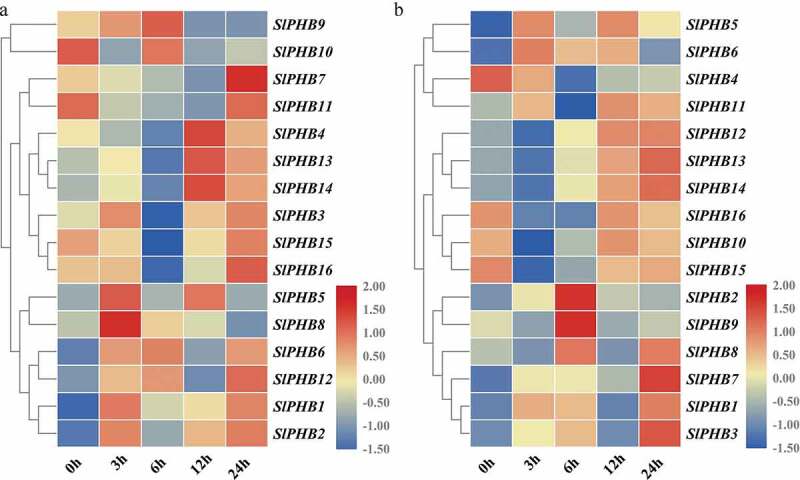
Abiotic stress-induced expression profile of *SlPHB* genes. (a) salt (b) drought (PEG) induced expression profile at 0 h, 3 h, 6 h, 12 h, and 24 h time points. A log2 transformed heatmap was generated using heatmapper program. Blue, white, and red color is corresponding to low, moderate, and high expressions. The genes were clustered by applying the Euclidean method.

To further investigate the role of *SlPHB* in tomato against abiotic stresses, the expression profile of *SlPHB* in response to salt and drought was analyzed at various time points. It was observed that under salt stress, the transcript abundance of *SlPHB9* was sharply increased at 3 h and peak at 6 h time point and subsequently declined at 12 h and 24 h time points. *SlPHB7* and *SlPHB11* had maximum transcript levels at 24 h while, *SlPHB4, SlPHB13*, and *SlPHB14* exhibited transcript abundance at 12 h time point. *SlPHB5* and *SlPHB8* induced only at 3 h after treatment but *SlPHB10* induced at 6 h time point (Fig. 6(a)). Under drought conditions, the majority of genes were expressed at the late time point (12 h and 24 h). *SlPHB2* and *SlPHB9* induced only at 6 h after treatment (Fig. 6(b)). In comparison, *SlPHB5, SlPHB13, SlPHB14, SlPHB15, SlPHB9*, and *SlPHB7* showed similar trends of expression under both drought and salinity stresses but *SlPHB4, SlPHB2*, and *SlPHB8* exhibited opposite trends under both stresses (Fig. 6). These results suggest that tomato *SlPHB* genes may play a key role in regulating abiotic stress responses.

### 3.7. *Phytohormone Induced Expression Profile Analysis of* SlPHBs *in Tomato*

**Figure 7.**
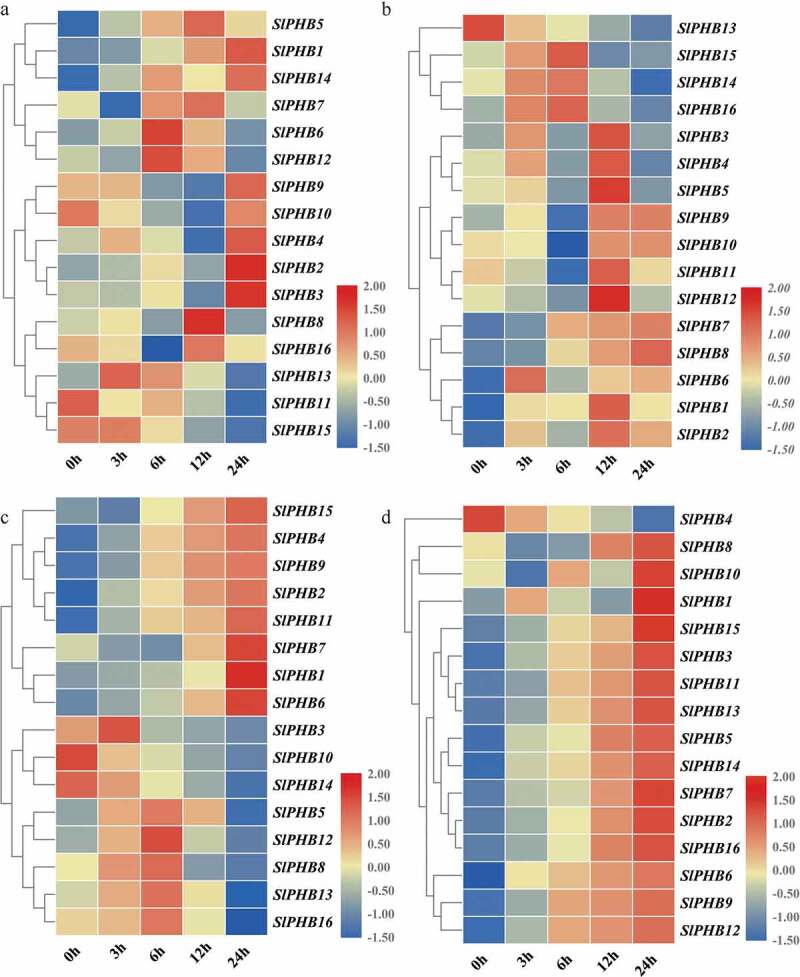
Phytohormone induced expression profile of *SlPHB* genes. (a) abscisic acid (ABA), (b) gibberellin (GA3), (c) auxin (IAA), (d) methyl jasmonate (MeJA) induced expression profile at 0 h, 3 h, 6 h, 12 h, and 24 h time points. A log2 transformed heatmap was generated using heatmapper program. Blue, white, and red color is corresponding to low, moderate, and high expressions. The genes were clustered by applying the Euclidean method.

To check the effectiveness of exogenous phytohormone application, the expression profile of tomato *SlPHB* under various hormones such as abscisic acid, gibberellin, auxin, and methyl jasmonate was examined. For ABA treatment, *SlPHB13* and *SlPHB15* were induced at 3 h time points while *SlPHB6* and *SlPHB12* were upregulated at 6 h after application with decreased expression in later time points. *SlPHB11* expression was downregulated upon treatment with ABA but *SlPHB8* and *SlPHB16* were induced only at 12 h after treatment. Moreover, *SlPHB9, SlPHB10, SlPHB4, SlPHB12*, and *SlPHB3* was upregulated at 24-time points (Fig. 7(a)). *SlPHB5, SlPHB14*, and *SlPHB16* transcript levels were sharply induced at 3 h interval and reach a maximum at 6 h time point but decreased in subsequent time intervals to GA3. *SlPHB3, SlPHB4, SlPHB5, SlPHB11, SlPHB12, SlPHB1*, and *SlPHB2* were induced with maximum transcript levels at 12 h after exposure to GA3 (Fig. 7(b)). The transcript abundance of *SlPHB7* and *SlPHB8* was increased temporally but *SlPHB13* expression was downregulated upon treatment with GA3. For auxin, *SlPHB10* and *SlPHB14* genes were downregulated after application but *SlPHB3* showed maximum transcript accumulation at 3 h point interval. *SlPHB5, SlPHB12, SlPHB8, SlPHB13*, and *SlPHB16* was upregulated with time and reached maximum expression at 6 h after treatment while, *SlPHB15, SlPHB4, SlPHB9, SlPHB2*, and *SlPHB11* expression levels were upregulated across 6 h to 24 h time points and showed maximum expression at 24 h interval (Fig. 7(c)). The *SlPHBs* exhibited a unique expression profile upon exposure to MeJA. It was observed that all the genes were upregulated temporally across all time intervals and have high transcript accumulation at 24 h time point except for *SlPHB4* (Fig. 7(d)). The data suggest that tomato *SlPHB* genes may play various important roles in cross-talk with different kinds of hormones signaling.

### 3.8. Subcellular Localization Assay

**Figure 8.**
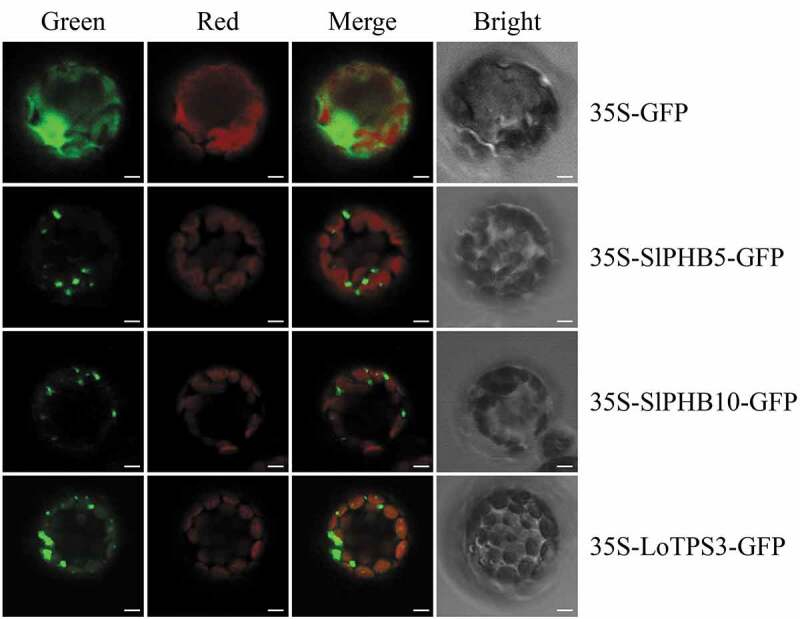
Subcellular localization images of SlPHB5 and SlPHB10 in Arabidopsis protoplasts. The full-length sequences of SlPHB5 and SlPHB10 were fused in the pro35S vector to generate p35S-SlPHBs/GFP constructs. The images were observed via confocal laser scanning microscopy. The LoTPS3 form *Lilium* ‘Siberia’ was used as red mitochondrial control for SlPHB5 and SlPHB10. The green, red, merged and BF represents the GFP fluorescence, chlorophyll autofluorescence, combined chlorophyll autofluorescence, and GFP fluorescence and bright field respectively. Scale bars 5 µm.

The amino acid sequence of SlPHB5 and SlPHB10 was submitted to the WoLFPSORT (https://wolfpsort.hgc.jp/) to predict subcellular localization. The predicted results showed that both SlPHB proteins were expressed in the mitochondria. To experimentally verify, full-length sequences of candidate SlPHB5 and SlPHB10 were fused to a GFP reporter gene and transferred to Arabidopsis protoplast (Fig. 8). Subcellular localization experiment results revealed that both proteins were localized in the mitochondria as predicted. LoTPS3 protein from Lilium Siberia was used as a positive control.^51^ Scale bar 5 µm.

## 4. Discussion

PHB, a highly conserved multigene family has been identified in many organisms from humans to various plant species playing essential roles in various aspects of growth and development. In plants, the PHB gene family has been reported from *Arabidopsis* (17), rice (19),^31^ *Glycine max* (24),^32^ and *Zea mays* with 16.^17^ However, no genome-wide identification of the PHB gene family has been reported in the tomato genome. In this study, a total of 16 PHB genes were identified in the tomato genome (Table 1). The tomato genome size (960Mb) is 7.68 folds of the Arabidopsis genome (125 Mb), 2.46 folds of rice (389 Mb) but 2.3 folds less of maize (2300 Mb) and 1.14 folds less than soybean (1100 Mb) genome. However, the number putative PHBs in the tomato genome even lower than *Arabidopsis* and rice^31^ but equal to reported in maize.^17^

Gene duplication either segmental or tandem plays an important role in the expansion of the genome. The expansion of the PHB gene family in *Arabidopsis*, rice, and soybean was caused by segmental duplication while tandem duplication was another cause of an increasing number of PHB genes in *Arabidopsis* but was absent in tomato. This implying that gene duplication of the PHB gene family in tomato was different from *Arabidopsis*. We have analyzed Ka/Ks values of three pairs of SlPHB gene duplication and found that tomato PHB genes undergo purifying selection (Table 2).

The PHB genes from fungi and mammals including humans were clustered in five phylogenetic clades. However, like *Arabidopsis*, rice,^31^ *Glycine max*,^32^ and *Zea mays*,^17^ tomato *SlPHBs* were also clustered in four clades. The genes sharing clades and subclades displayed a similar gene structure and conserved motifs patterns. PHB genes are involved in various aspects of plant growth and development. In this study, *cis*-regulatory sequences were predicted. It was observed that tomato *SlPHB* genes contained various development, abiotic stress, and phytohormone responsive elements in their promoter regions (Fig. 4). It has been well documented that PHB genes involved in leaf yellowing, hormone signal transduction pathways, and abiotic stress responses. For example, *Arabidopsis AtPHB3/4* causes proliferation of root and shoot tissues.^24^ Similarly, *petunia* PHBs, tobacco *NbPHB1/2* promote leaf senescence.^25,26^ In this study, the expression profile of *SLPHBs* in various parts of tomato plant was also investigated. Tomato *SlPHB* genes showed diverse expression patterns in different parts such as *SlPHB4* and *SlPHB10* was expressed in flower and root tissues, respectively. Two genes (*SlPHB8, SlPHB9*) were highly expressed in 2 cm fruit while *SlPHB5, SlPHB14*, and *SlPHB15* showed increasing expression pattern with the fruit development stages (Fig. 5). These results suggest the crucial role of *SlPHB* genes in development of these organs in tomato plant.

In this study, *cis*-regulatory elements involved in diverse signaling pathways were identified. Most PHBs contain *cis*-regulatory elements involved in ABA, GA, JA, and ethylene. In addition, *cis*-elements involved in abiotic stresses, such as MBS (MYB binding site involved in drought-inducibility), LTR (low-temperature responsiveness element), HSE (heat stress responsiveness element), were also observed in the promoter regions of *SlPHB* genes (Fig. 4). In *Glycine max*, most of PHBs contained numerous hormone-responsive, development and stress-related *cis*-regulatory elements in the GmPHB promoters.^32^ It was observed that the expression of *SlPHB* genes was altered under these stresses. For salt treatment, *SlPHB5, SlPHB8, SlPHB9*, and *SlPHB10* were upregulated at early time points (3 h and 6 h) while, *SlPHB7, SlPHB11, SlPHB4, SlPHB13, SlPHB14*, and *SlPHB12* were induced at 12 h and 24 h after treatment (Fig. 6(a)). Similar response was observed in *Arabidopsis*, where PHBs were involved in abiotic stimulus and phytohormones functioning.^16,^^29^ *SlPHB2* and *SlPHB9* genes were induced under drought at 6 h time point but *SlPHB4* was downregulated (Fig. 6(b)). *SlPHB1, SlPHB14, SlPHB9, SlPHB10, SlPHB4*, and *SlPHB3* were upregulated after 24 h exposure to ABA but *SlPHB11* and *SlPHB15* were downregulated upon exposure (Fig. 7(a)). Moreover, *SlPHB13, SlPHB14, SlPHB15*, and *SlPHB16* were suppressed in late intervals of GA3 exposure but the rest of the genes were upregulated (Fig. 7(b)). *SlPHB10* and *SlPHB14* were downregulated after auxin application but, *SlPHB3* was induced after 3 h of treatment. *SlPHB7, SlPHB1*, and *SlPHB6* exhibited maximum expression at a 24 h time point (Fig. 7(c)). For MeJA treatment, all the genes were induced sharply along with all the time points and peaked at 24 h after treatment except for *SlPHB4*, which was suppressed upon exposure to MeJA (Fig. 7(d)). Likewise, *Atphb3* mutant was highly responsive to ethylene in etiolated seedlings. One Arabidopsis prohibitin (At5g64870) was down-regulated under some hormones (GA, MeJA and ABA), while highly upregulated under salt, drought and cold treatment.^31^ In *Capsicum annum*, hypersensitive-induced reaction (HIR) proteins (PHB encoding proteins), such as *CaHIR1*, maize *ZmHIR1*-3, barley *HvHIR1*/3 and *AtHIR1*-3 were induced under abiotic stresses.^57–59^ Our findings are in line with previous studies that *PHB* genes showed differential expression pattern under different development stages as well as under different stimulus.^5,25,26,30,31^ The above-mentioned findings highlighted the potential diverse role of PHB genes.

## 5. Conclusion

In short, this study provides knowledge about the PHB gene family in the tomato genome. All the identified SlPHBs were clustered in four clades according to the phylogenetic tree. The gene structure and conserved motifs distribution patterns in each clade validated the phylogenetic classification of tomato SlPHBs. *Cis*-regulatory sequences prediction in combination with complex regulation of tomato PHB genes family expression against salinity, drought, and various phytohormones such as ABA, IAA, GA, and MeJA provide a foundation for further functional characterization of these genes in tomato and other plant species.

## Supplementary Material

Supplemental Material

Supplemental Material
