# Supplementary material for: The prohibitins (PHB) gene family in tomato: Bioinformatic identification and expression analysis under abiotic and phytohormone stresses
Source: GM Crops Food. 2021 Mar 8;12(1):535–50. doi: 10.1080/21645698.2021.1872333 (PMC8820253; doi:10.1080/21645698.2021.1872333)
Supplement: Supplemental Material [file KGMC_A_1872333_SM0413.docx]

**Supplementary Table 2:** List of primers used in the experiments

| **Gene** |  | **Upstream / downstream primers（5′-3′）** | **Purpose** |
| --- | --- | --- | --- |
| Solyc01g010770.2.1 | PHB1-F | GAGAAGCTGCATTTGAGAA | RT-qPCR |
|  | PHB1-R | CACTAGTAGAGAAGCCAATAAC | RT-qPCR |
|  |  |  |  |
| Solyc01g089910.2.1 | PHB2-F | GTTGACGGAACAAGTAGTG | RT-qPCR |
|  | PHB2-R | TGGATGAGGCTGGATTAG | RT-qPCR |
|  |  |  |  |
| Solyc03g005420.1.1 | PHB3-F | GTAACCACCCAAAGACAAG | RT-qPCR |
|  | PHB3-R | ACTCAGCCTCTCGTATTG | RT-qPCR |
|  | **Gene** |  |  |
| Solyc03g007190.2.1 | PHB4-F | GAGCAGATTCAGGCTTATG | RT-qPCR |
|  | PHB4-R | CATCAGTATGTGCTCGATATTA | RT-qPCR |
|  |  |  |  |
| Solyc03g080050.2.1 | PHB5-F | AAGCAGTCCTCTCTTCTATC | RT-qPCR |
|  | PHB5-R | CTTCCCAAATCGCTCAAC | RT-qPCR |
|  |  |  |  |
| Solyc03g113220.2.1 | PHB6-F | CAGACACTCATCGTTGATATAG | RT-qPCR |
|  | PHB6-R | CCTAATCCTGAGAGATACTTAGA | RT-qPCR |
|  |  |  |  |
| Solyc03g117250.2.1 | PHB7-F | CAAGGCTACTGTCGAATATG | RT-qPCR |
|  | PHB7-R | GCTGCCGAGTATAGAGTT | RT-qPCR |
|  |  |  |  |
| Solyc05g012340.2.1 | PHB8-F | CGGAGACTTCACTCCTATT | RT-qPCR |
|  | PHB8-R | CACCTCCTCTCCAATAGAC | RT-qPCR |
|  |  |  |  |
| Solyc05g051510.2.1 | PHB9-F | TAGGGCTGAAGGAGAAAG | RT-qPCR |
|  | PHB9-R | TTCTGTTTGGGCAAGTAAG | RT-qPCR |
|  |  |  |  |
| Solyc06g065850.2.1 | PHB10-F | TTCCAGGACACGAGTATTT | RT-qPCR |
|  | PHB10-R | CCTTCTCCTTCCCTTTGT | RT-qPCR |
|  |  |  |  |
| Solyc06g071050.2.1 | PHB11-F | GAGAATGTGAAGAGAGCTATG | RT-qPCR |
|  | PHB11-R | CTGTCGTGCAATACCTAAC | RT-qPCR |
|  |  |  |  |
| Solyc06g073030.1.1 | PHB12-F | GAAGCTGAAGCCATTCTC | RT-qPCR |
|  | PHB12-R | CAGGATCAGAGACATTTGTAG | RT-qPCR |
|  |  |  |  |
| Solyc10g008140.2.1 | PHB13-F | TATCCAGAAGGGACACAC | RT-qPCR |
|  | PHB13-R | CACCAAGAGTTCGGTAAAC | RT-qPCR |
|  |  |  |  |
| Solyc11g010190.1.1 | PHB14-F | CAACCAAGCAGCAGTATC | RT-qPCR |
|  | PHB14-R | GGGACGAGGAAATGAGTA | RT-qPCR |
|  |  |  |  |
| Solyc11g013260.1.1 | PHB15-F | GATGAAAGCTGAGCAAGAG | RT-qPCR |
|  | PHB15-R | TAGGCAAGTAAGCAACATTAG | RT-qPCR |
|  |  |  |  |
| Solyc12g005500.1.1 | PHB16-F | CCGTATTCGTGGTGTTAAAG | RT-qPCR |
|  | PHB16-R | CACCAAGTGATCGGTAAATAG | RT-qPCR |
|  |  |  |  |
| Solyc03g080050 | PHB16-F | TTACAATTACAGTCGACTAGTG ATGAACACGGCGAGATCG | GFP |
|  | PHB16-R | CCTTGCTCACCATGGATCGATC GTCATCCTTTTTTTGGCT | GFP |
|  |  |  |  |
| Solyc06g065850 | PHB16-F | TTACAATTACAGTCGACTAGTG ATGTATAGAGTTGCAA | GFP |
|  | PHB16-R | CCTTGCTCACCATGGATCGATC TTGCATTATTTCTTGAGG | GFP |
|  |  |  |  |
| Solyc03g080050 | PHB16-F | ATGAACACGGCGAGATCGAACTC | Cloning |
|  | PHB16-R | CGCTGCAAAGCCAAAAAAAGGATG | Cloning |
|  |  |  |  |
| Solyc06g065850 | PHB16-F | ATGTATAGAGTTGCAAAAGCATC | Cloning |
|  | PHB16-R | CCTCAAGAAATAATGCAATGA | Cloning |
