# Supplementary material for: The prohibitins (PHB) gene family in tomato: Bioinformatic identification and expression analysis under abiotic and phytohormone stresses
Source: GM Crops Food. 2021 Mar 8;12(1):535–50. doi: 10.1080/21645698.2021.1872333 (PMC8820253; doi:10.1080/21645698.2021.1872333)
Supplement: Supplemental Material [file KGMC_A_1872333_SM0395.docx]

| **Table S1. List of miRNAs predicted by psRNATarget to target the tomato PHB genes** | | | | | | | | | | | |
| --- | --- | --- | --- | --- | --- | --- | --- | --- | --- | --- | --- |
| miRNA_Acc. | Target_Acc. | Expectation | UPE | miRNA_start | miRNA_end | Target_start | Target_end | miRNA_aligned_fragment | Target_aligned_fragment | Inhibition | Multiplicity |
| Sly-miR397 | SlPHB13 | 2.5 | 14.597 | 1 | 21 | 513 | 533 | UCAUUGAGUGCAGCGUUGAUG | CUUCAACAUUGCACUUGAUGA | Cleavage | 1 |
| Sly-miR396 | SlPHB15 | 3 | 20.23 | 1 | 21 | 544 | 564 | UUCCACAGCUUUCUUGAACUG | GAGUUUUCGAAAGCUGUGGAG | Cleavage | 1 |
| Sly-miR4239 | SlPHB3 | 3 | 17.007 | 1 | 21 | 774 | 794 | UUUGUUAUUUUCGCAUGCUCC | UCAGCAGGCGAAGAUGGCAGA | Cleavage | 1 |
| Sly-miR869 | SlPHB7 | 3 | 10.713 | 1 | 21 | 1526 | 1546 | AUUGGUUCAAUUCUGGUGUUG | UGAAACUUGAAUUGAAUCAAU | Cleavage | 1 |
